# Epidemiologic comparison of the first and second waves of coronavirus disease in Babol, North of Iran

**DOI:** 10.22088/cjim.11.0.544

**Published:** 2020

**Authors:** Seyed Farzad Jalali, Mostafa Ghassemzadeh, Simin Mouodi, Mostafa Javanian, Mehdi Akbari Kani, Reza Ghadimi, Ali Bijani

**Affiliations:** 1 Department of Cardiology, Babol University of Medical Sciences, Babol, Iran; 2 Department of Dermatology, Babol University of Medical Sciences, Babol, Iran; 3 Social Determinants of Health Research Center, Health Research Institute, Babol University of Medical Sciences, Babol, Iran; 4 Infectious Diseases and Tropical Medicine Research Center, Health Research Institute, Babol University of Medical Sciences, Babol, Iran; 5 Babol University of Medical Sciences, Babol, Iran

**Keywords:** Coronavirus, Epidemiology, Disease outbreaks

## Abstract

**Background:**

A few studies compared the epidemiologic features of the first and second waves of coronavirus disease 2019 (COVID-19) outbreak. This research was carried out to compare the 1^st^ and the 2^nd^ waves of the epidemics in the northern Iran.

**Methods:**

In this observational research, demographic, clinical and laboratory characteristics of the patients with COVID-19, admitted to four government hospitals affiliated to Babol University of Medical Sciences during the 1^st^ and the 2^nd^ waves of COVID-19 epidemics have been compared. The period from May 21, 2020 to September 21, 2020 was considered as the second wave of the epidemics while from February 19, 2020 to May 20, 2020, as the first wave of the outbreak in this region.

**Results:**

Out of 6691 total hospitalized cases, 4374 (65.37%), including 1532 (49.6%) people in the first wave and 2842 (78.9%) in the second wave had RT-PCR test for disease confirmation. Among those who were examined with RT-PCR test, 2322 patients (53.1%) including 728 (31.4%) persons in the first wave and 1594 (68.6%) in the second wave were positive for SARS-CoV-2 RNA. 414 (56.9%) of the confirmed cases in the first wave and 767 (48.1%) in the second wave were males (p<0.001). Gastrointestinal symptoms were more incidental in the second wave of the disease. However, severe respiratory conditions were more common during the first wave (p<0.001). Crude mortality rate was lower in the second wave of the outbreak (p<0.001).

**Conclusion:**

Different epidemiologic characteristics were found in the second wave of COVID-19 outbreak in comparison with the first wave of the epidemics in northerrn Iran.

The new coronavirus disease called coronavirus disease 2019 (COVID-19), which started in late 2019 and caused a pandemic around the world, has affected people, organizations and countries worldwide, and no one still knows to what extent and for how long it will continue and what will be the future of this disease in different regions (1). Based on the reports collected from the affected countries, the World Health Organization has reported a total of 37,568,843 laboratory confirmed cases of the disease including 1,077,508 deaths as of October 12, 2020. Iran is one of the countries affected by the COVID-19 outbreak and a significant number of confirmed cases and deaths related to the disease has been reported in Iran so far. The first recorded case of the disease was on February 19, 2020 in the city of Qom and subsequently in a short time, have been reported from other provinces of the country (2, 3). Based on the evidence reported, at some points in time, Iran ranked as the first countries in the world in terms of the crude number of patients with COVID-19, or the number of improved patients and deaths due to the disease (2).

Iranian Ministry of Health, Treatment and Medical Education has reported a total of 504,281 people that were infected with the virus and 28,816 people died until October 13, 2020. In Babol, as an overgrown city located in the North of Iran, near the Caspian Sea, the first COVID-19 patient was reported on February 20, 2020. This city experienced two waves of the disease epidemic until October 2020. 

A review of literatures describing the clinical signs and laboratory findings of 100 confirmed patients with COVID-19, aged 18 years and over admitted to the government hospitals of this city during the first wave of the disease showed that the average age of patients was 60.12 years; half of them were females; and 50% of patients had another comorbid disease, especially, diabetes mellitus, high blood pressure, coronary heart diseases, and chronic kidney disorders, respectively. In addition, the most incidental clinical manifestations of the patients were reported as anorexia, dry cough, dyspnea, fever and fatigue, respectively (4).

 In this city, since May 21, 2020, coincided with the beginning of warm days of the year in this area, the number of confirmed cases of COVID-19 and referral rate to the governmental hospitals because this disease has increased again, and the second wave of the epidemic started. This research was carried out to describe the epidemiologic features of the disease in the second wave and to compare with the first wave of the COVID-19 outbreak. Of course, the comparison of different epidemiological dimensions of these two waves can be effective in the health policy-making and planning for the prevention and control of disease in the northern region of Iran (1, 5-7). 

## Methods

In this observational study, the demographic, clinical and laboratory characteristics of the patients admitted to the four government hospitals affiliated to Babol University of Medical Sciences, as suspected or confirmed cases with COVID-19, during the period from May 21, 2020 to September 21, 2020 (considered as the second wave of the epidemic in this region) have been compared with similar characteristics of the patients admitted in these hospitals from February 19, 2020 to May 20, 2020 (as the first wave of the epidemic). Collected data included the patient's age and gender, clinical symptoms, underlying disorders, need to ICU admission and tracheal intubation, and the final outcome of the disease at the time of hospital discharge. The source for data collection was the hospital-based data bank of the COVID-19 patients, named as Medical Care Monitoring Center (MCMC) which has been designed by the Ministry of Health, Treatment and Medical Education of Iran, and is used in the government hospitals of the country. The patients whose nasopharyngeal or oropharyngeal specimens were positive for SARS-CoV-2 RNA using real-time polymerase chain reaction (RT-PCR) have been considered as confirmed cases; and persons with defined clinical, epidemiologic and laboratory criteria, without confirmatory tests have been classified as suspected patients (8). Data analysis was performed using SPSS-18 software package. Chi-square and logistic regression tests were used for data analysis. A p-value less than 0.05 was considered as the significance level. This research has been approved by the Ethics Committee of Babol University of Medical Sciences, Iran with reference ID: IR.MUBABOL.HRI.REC.1399.119. 

## Results

Totally, 3091 confirmed and suspected patients were admitted in the four mentioned hospitals during the first wave of COVID-19 epidemic and 3600 individuals were admitted during the second wave. It shows that the crude number of patients admitted during the second wave of the disease was 1.41 (95% CI: 1.25-1.60) times more than the hospitalized people in the first wave. Mean age of patients in the first and second waves was 56.84±18.29 and 53.60±23.05 years, respectively (p<0.001). Out of 6691 total cases, 4374 (65.37%), including 1532 (49.6%) people in the first wave and 2842 (78.9%) in the second wave had RT-PCR test for disease confirmation. 

**Figure 1:**
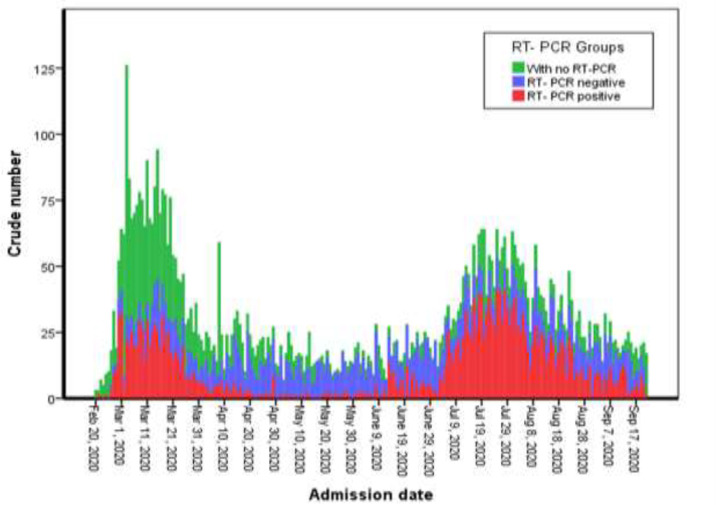
Distribution of hospitalized patients during the two waves of COVID-19 outbreaks in Babol, North of Iran

Among those who were examined with RT-PCR test, 2322(53.1%) patients including 728 (31.4%) persons in the first wave and 1594 (68.6%) in the second wave were positive for SARS-CoV-2 RNA. Crude number of hospitalized patients during the two waves of COVID-19 outbreak was divided as individuals with positive and negative RT-PCR test and those with no confirmatory test have been presented in figure 1. 

Among the 2322 confirmed cases with COVID-19, 1181 (50.9%) individuals were males and 1141 (49.1%) were females; 414 (56.9%) of the confirmed cases in the first wave were males; however, this proportion for male gender was 767 (48.1%) in the second wave (p<0.001). 

**Figure 2:**
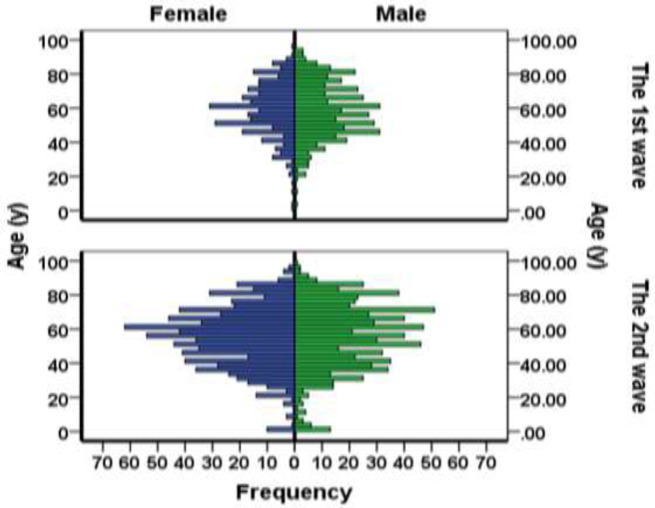
Age and sex distribution of patients with confirmed COVID-19 during the two waves of the disease in Babol, North of Iran

Age and sex distribution of confirmed cases has been presented in figure 2. One-hundred and twenty (7.5%) individuals of the confirmed cases in the second wave, and 75 (10.3%) in the first wave reported an obvious close contact with another well-known patient with coronavirus disease. 

**Figure 3:**
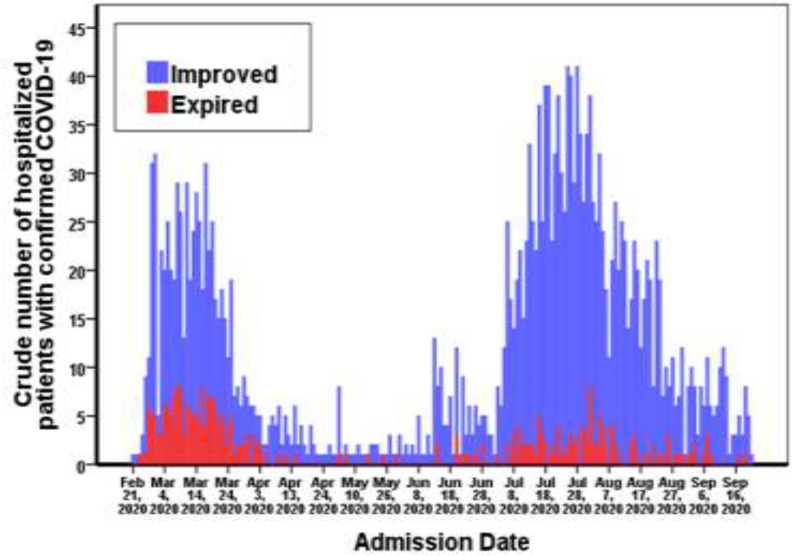
Distribution of the improved and expired patients with confirmed COVID-19 during the two waves of the disease, Babol, North of Iran

Clinical manifestations that were more common among patients with confirmed COVID-19 during the two waves of the disease has been summarized in Table 1. This table shows that fever (OR=1.98; 95% CI: 1.66-2.36; p<0.001), myalgia (OR=1.43; 95% CI: 1.17-1.76; P=0.001), and gastrointestinal (GI) symptoms such as diarrhea (OR=5.16; 95% CI: 2.07-12.83; p<0.001), taste disorders (OR=3.53; 95% CI: 1.06-11.81; P=0.029), nausea (OR=2.97; 95% CI: 1.62-5.45; p<0.001), vomiting (OR=2.56; 95% CI: 1.26-5.17; P=0.007) and abdominal pain (OR=2.36; 95% CI: 1.06-5.25; P=0.031) were more incidental in the second wave of the disease compared to the first one. However, severe respiratory conditions and need to ICU care (p<0.001) or tracheal intubation (p<0.001) were observed with higher rate during the first wave of the disease. Underlying disorders comorbid to coronavirus disease among confirmed cases have been presented in table 2. This table represents diabetes mellitus, cardiovascular disorders, high blood pressure, chronic renal disorders, malignancies, asthma and other respiratory disorders as the most common comorbidities in hospitalized patients with COVID-19, respectively. Also, during the second wave, cardiovascular disorders P=0.033), hypertension (p<0.001), chronic renal disorders (P=0.044), malignancies (P=0.022), and opium use (P=0.046) were more prevalent among the study population, compared to the first wave. Totally, 127 (8.0%) of patients with confirmed COVID-19 during the 2^nd^ wave, and 170 (23.4%) during the 1^st^ wave expired. Crude mortality rate was lower in the second wave of the disease (p<0.001). During the second wave, the highest mortality rate was in the age group of 80-89 (n=32; 25.2%), 60-69 (n=30; 23.6%) and 50-59 (n=27; 21.3%) years, respectively; and during the first wave, the age-groups of 60-69 (n=41; 24.1%), 70-79 (n=41; 24.1%) and 50-59 (n=38; 22.4%) had the highest mortality rate. Distribution of the improved and expired patients during the two waves of the disease has been presented in figure 3.

**Table 1:** Distribution of clinical features in patients with confirmed COVID-19 during the two waves; North of Iran

**Clinical features**	**The 1** ^st^ ** wave n=728** **N(%)**	**The 2** ^nd^ ** wave n=1594** **N(%)**	**Total** **n=2322 N(%)**	**P-value**	**Crude Odds Ratio for the 2** ^nd^ ** wave/the 1** ^st^ ** wave** **(95% CI)**
Fever (measured or subjective)	330 (45.3)	990 (62.1)	1320 (56.8)	<0.001	1.98 (1.66-2.36)
Dry cough	391 (53.7)	741 (46.5)	1132 (48.8)	0.001	0.75 (0.63-0.89)
Respiratory distress	430 (59.1)	635 (39.8)	1065 (45.9)	<0.001	0.46 (0.38-0.55)
Myalgia	161 (22.1)	461 (28.9)	622 (26.8)	0.001	1.43 (1.17-1.76)
Nausea	12 (2.2)	99 (6.2)	111 (5.2)	<0.001	2.97 (1.62-5.45)
Diarrhea	5 (0.9)	72 (4.5)	77 (3.6)	<0.001	5.16 (2.07-12.83)
Vomiting	9 (1.6)	65 (4.1)	74 (3.5)	0.007	2.56 (1.26-5.17)
Impaired consciousness	26 (3.6)	43 (2.7)	69 (3.0)	0.250	0.75 (0.46-1.23)
Abdominal pain	7 (1.3)	47 (2.9)	54 (2.5)	0.031	2.36 (1.06-5.25)
Anorexia	15 (2.7)	29 (1.8)	44 (2.1)	0.195	0.66 (0.35-1.24)
Taste disorders	3 (0.4)	23 (1.4)	26 (1.1)	0.029	3.53 (1.06-11.81)
Olfactory disorders	3 (0.4)	16 (1.0)	19 (0.8)	0.143	2.45 (0.71-8.42)
Convulsion	3 (0.4)	11 (0.7)	14 (0.6)	0.423	1.68 (0.47-6.03)
Need to ICU care	129 (17.7)	140 (8.8)	269 (11.6)	<0.001	0.45 (0.35-0.58)
Need to tracheal intubation	168 (23.1)	57 (3.6)	225 (9.7)	<0.001	0.12 (0.09-0.17)

**Table 2:** Comorbid disorders in hospitalized patients with confirmed COVID-19, North of Iran

**Underlying disorders comorbid to coronavirus disease**	**The 1** ^st^ ** wave n=728** **N(%)**	**The 2** ^nd^ ** wave n=1594** **N(%)**	**Total** **n=2322 N(%)**	**P-value**	**Crude Odds Ratio for the 2** ^nd^ ** wave/the 1** ^st^ ** wave** **(95% CI)**
Diabetes mellitus	154 (21.2)	363 (22.8)	517(22.3)	0.384	1.10 (0.89-1.36)
Cardiovascular disorders	157 (21.6)	284 (17.8)	441(19.0)	0.033	0.79 (0.63-0.98)
Hypertension	46 (6.3)	275 (17.3)	321(13.8)	<0.001	3.09 (2.23-4.28)
Chronic renal disorders	26 (3.6)	88 (5.5)	114 (4.9)	0.044	1.58 (1.01-2.47)
Malignancies	8 (1.1)	41 (2.6)	49 (2.1)	0.022	2.38 (1.11-5.10)
Asthma	20 (2.7)	27 (1.7)	47 (2.0)	0.094	0.61 (0.34-1.10)
Other respiratory disorders (Except Asthma)	16 (2.2)	22 (1.4)	38 (1.6)	0.150	0.62 (0.33-1.19)
Opium abuse	3 (0.4)	21 (1.3)	24 (1.0)	0.046	3.22 (0.96-10.84)
Chronic neurologic disorders	8 (1.1)	20 (1.3)	28 (1.2)	0.750	1.14 (0.50-2.61)
Smoking	6 (0.8)	12 (0.8)	18 (0.8)	0.854	0.91 (0.34-2.44)
Chronic hematopoietic disorders	6 (0.8)	9 (0.6)	15 (0.6)	0.469	0.68 (0.24-1.93)
Chronic liver diseases	1 (0.1)	7 (0.4)	8 (0.3)	0.250	3.21 (0.39-26.11)
Chronic immunologic disorders (Except HIV/AIDS)	3 (0.4)	6 (0.4)	9 (0.4)	0.898	0.91 (0.23-3.67)
HIV/AIDS	2 (0.3)	1 (0.1)	3 (0.1)	0.187	0.23 (0.02-2.52)
Other comorbid disorders	76 (10.4)	138 (8.7)	214 (9.2)	0.168	0.81 (0.61-1.09)

## Discussion

This research describes the two waves of COVID-19 epidemic in the northern region of Iran. Our results showed that the first wave of the disease, which occurred during the relatively cold months of the year, was associated with more deaths; however, the second wave of the disease, which began at the start of the relatively warm months of the year, although more patients were hospitalized during the wave, mortality rate dropped significantly. The first wave of the epidemic began when no standard and comprehensive protocol was published for treatment of patients, worldwide; and scientific evidence was mainly the results of experiences from limited studies, usually in small sample size populations or small study areas, that presented various treatment approaches to health-care professionals (9). In addition, at that time, widespread RT-PCR assays for precise diagnosis of COVID-19 in symptomatic patients were not easily available; in this way, fewer patients have been examined using RT-PCR test during the first wave of the disease. Babol University of Medical Sciences has established the first RT-PCR laboratory since March 5, 2020, and also improved the instruments and trained staff to perform this assay over time, therefore, case confirmation has increased in the second wave compared to the first one. Another factor that might have impact on final outcome of the patients during the two waves is the availability of different treatment facilities in the state hospitals of the region (10). During the first wave, more patients with COVID-19 required respiratory support (11, 12); furthermore, age, gender, disease severity and comorbidities are other important factors that can justify the observed difference in mortality rate during these two waves (13-15). 

Mean age was lower in the second wave compared to the first one. A systematic review and meta-analysis reported a median age of 46.2 years among the patients with a confirmed COVID-19 diagnosis (16). Although the exact cause for the difference of the patients' age between the two waves is unknown, it has been hypothesized that older adults have a further risk of SARS-CoV-2 infection, disease-related hospitalization and more severe complications (4, 17, 18). This can be associated to identify older adults in the first wave of the outbreak and younger adults in the later phases of the epidemic. 

In our study, women were affected with a higher proportion than men during the second wave of the disease, while during the first wave, men showed further vulnerability to the disease. It may be due to immunological, lifestyle behaviors such as smoking, health related self-care, or other factors that can potentially change the gendered impacts of the epidemic (19, 20). However, our current data on sex differences is incomplete. Gastrointestinal manifestations were more common in the second wave. Literature review shows that GI features can be present in more than 25% of patients with COVID-19 (21). The most incidental GI symptoms in these patients have been reported as diarrhea, nausea and/or vomiting and abdominal pain (21-24). It is important to be aware of gastrointestinal symptoms in both adult and pediatric populations. Be unfamiliar to these presentations can lead to delay in early diagnosis and treatment of patients with COVID-19, and cause serious complications. 

Diabetes mellitus, cardiovascular disorders, hypertension, chronic renal disorders, malignancies, asthma and other respiratory disorders were the most common comorbidities in hospitalized patients with confirmed COVID-19; and all of these comorbidities were more prevalent in the second wave of the epidemic compared to the first wave. A recent meta-analysis revealed that medical comorbidities can lead to higher incidence of serious events such as ICU admission, pneumonia, acute respiratory distress syndrome, mechanical ventilation, and death in patients with COVID-19; for example, presence of chronic respiratory disorders, chronic kidney diseases, cardiovascular diseases, and diabetes mellitus caused a 6.6, 5.3, 4.5, and 3.07 times higher risk of developing serious events in COVID 19 patients, respectively (25). The most important strength point of this study is its novelty on describing the two waves of COVID-19 outbreak. Up to this writing, the number of studies that described more than one wave of the COVID-19 outbreak was very limited (26). We did not report details of laboratory and imaging characteristics of the patients and this can be presented as a limitation of this research. 

In conclusion, different epidemiologic characteristics were observed in the second wave of COVID-19 outbreak in comparison with the first wave of the epidemic in the northern region of Iran. 
